# Validation of a Commercial Automated Body Condition Scoring System on a Commercial Dairy Farm

**DOI:** 10.3390/ani9060287

**Published:** 2019-05-29

**Authors:** Israel L. Mullins, Carissa M. Truman, Magnus R. Campler, Jeffrey M. Bewley, Joao H. C. Costa

**Affiliations:** 1 Department of Animal and Food Sciences, University of Kentucky, Lexington, KY 40546, USA; ilmu222@g.uky.edu (I.L.M.); carissa.truman@gmail.com (C.M.T.); magnus.campler@uky.edu (M.R.C.); 2 Alltech, Inc., Nicholasville, KY 40356, USA; jbewley@alltech.com

**Keywords:** automation, precision dairy farming, BCS camera, dairy management

## Abstract

**Simple Summary:**

The evaluation and implementation of an automated body condition scoring technology for dairy cattle. Body condition scoring in cattle is an effective tool to assess body reserves of individual animals. On-farm body condition scoring requires training and time to appropriately evaluate the animals. The aim of this study was to evaluate the implementation of an automated body condition scoring technology compared to conventional manual scoring. We found that the automated body condition scoring technology was highly correlated with manual scoring. The system was accurate for a body condition scoring (BCS) between 3.0 and 3.75, with a lower error rate compared to the standard detection threshold of 0.25 for manual scoring. However, the system was found to be in a different range of scores and was inaccurate at determining under- and over-conditioned cattle compared to manual scoring.

**Abstract:**

Body condition scoring (BCS) is the management practice of assessing body reserves of individual animals by visual or tactile estimation of subcutaneous fat and muscle. Both high and low BCS can negatively impact milk production, disease, and reproduction. Visual or tactile estimation of subcutaneous fat reserves in dairy cattle relies on their body shape or thickness of fat layers and muscle on key areas of the body. Although manual BCS has proven beneficial, consistent qualitative scoring can be difficult to implement. The desirable BCS range for dairy cows varies within lactation and should be monitored at multiple time points throughout lactation for the most impact, a practice that can be hard to implement. However, a commercial automatic BCS camera is currently available for dairy cattle (DeLaval Body Condition Scoring, BCS DeLaval International AB, Tumba, Sweden). The objective of this study was to validate the implementation of an automated BCS system in a commercial setting and compare agreement of the automated body condition scores with conventional manual scoring. The study was conducted on a commercial farm in Indiana, USA, in April 2017. Three trained staff members scored 343 cows manually using a 1 to 5 BCS scale, with 0.25 increments. Pearson’s correlations (0.85, scorer 1 vs. 2; 0.87, scorer 2 vs. 3; and 0.86, scorer 1 vs. 3) and Cohen’s Kappa coefficients (0.62, scorer 1 vs. 2; 0.66, scorer 2 vs. 3; and 0.66, scorer 1 vs. 3) were calculated to assess interobserver reliability, with the correlations being 0.85, 0.87, and 0.86. The automated camera BCS scores were compared with the averaged manual scores. The mean BCS were 3.39 ± 0.32 and 3.27 ± 0.27 (mean ± SD) for manual and automatic camera scores, respectively. We found that the automated body condition scoring technology was strongly correlated with the manual scores, with a correlation of 0.78. The automated BCS camera system accuracy was equivalent to manual scoring, with a mean error of −0.1 BCS and within the acceptable manual error threshold of 0.25 BCS between BCS (3.00 to 3.75) but was less accurate for cows with high (>3.75) or low (<3.00) BCS scores compared to manual scorers. A Bland–Altman plot was constructed which demonstrated a bias in the high and low automated BCS scoring. The initial findings show that the BCS camera system provides accurate BCS between 3.00 to 3.75 but tends to be inaccurate at determining the magnitude of low and high BCS scores. However, the results are promising, as an automated system may encourage more producers to adopt BCS into their practices to detect early signs of BCS change for individual cattle. Future algorithm and software development is likely to increase the accuracy in automated BCS scoring.

## 1. Introduction 

Body condition scoring (BCS) is a tool traditionally used to assess body reserves of individual animals [1]. The determination of BCS of cattle is conducted through examination of fat across various key points of the body such as the withers, hooks, tail head, and pins [2]. Although body weight provides valuable information, an accurate body condition of cattle is hard to determine solely through body weight as cattle vary greatly in skeletal size, udder, and gut fill [3]. Moreover, cattle are at increased risk of body condition loss and disease during the transition period [4,5,6] with the puerperal period of dairy cattle being the key period where management decisions play a large role in maintaining herd health [7,8]. Monitoring body condition before calving is of equal importance to pre-calving feeding management, as both are incredibly important for establishing immunocompetence post-calving [9].

Individual cattle monitoring and management decisions to stabilize BCS can be a mitigating factor in cattle at risk of contracting disease during the transition period [10]. Transition cattle with low BCS are at a greater risk to develop endometritis [11], and cattle with a BCS lower than 2.50 are at greater risk for developing lameness [12]. Patterns of BCS loss have shown to negatively impact conception and reproduction rates [13,14] and cattle health such as dystocia [7], ketosis [15] and metritis [16]. Fluctuations in BCS can alter production outcomes and impact financial stability for producers, as losses and gains in BCS throughout the transition period and lactation period have shown to be correlated to milk yield output [17]. Cattle that have greater loss of BCS early in lactation tend to be high producing cows [17] while lactating cattle that maintain condition or experience limited condition loss have been linked to lower milk yields [18], but this is not always the case [19]. However, cattle with a low BCS at calving produce less milk, and cattle that calve at a high BCS are more likely to contract periparturient metabolic disorders [20]. Additionally, it has previously been reported that cattle with a high BCS during the lactation period tend to have higher lactose and fat content in the milk compared to cattle with BCS of less than 2.50 [21,22]. Thus, body condition scores are often the key management tool for producers to help make production and nutrition management decisions, especially for individual cattle at risk of transition diseases that ultimately may impact animal welfare and farm economics. 

Despite the importance of monitoring body condition in cattle, a survey showed that only 36% of the herds surveyed implemented BCS into their management practices [23]. Farms that do implement BCS frequently rely on subjective measurements dependent on training and accuracy of farm staff, adding to the challenge of accurately and consistently determining BCS in cattle. Interestingly, the use of precision technology has increased in the dairy industry in recent years. For instance, estrus detection, milking, and other traditionally manual tasks on a dairy operation can now be completed with the use of precision technology [24]. Moreover, a survey study found that 47 out of 103 (46%) respondents utilized BCS on their farms, with 26 out of 103 (25%) using nutritionists, 12 out of 103 (12%) using farm staff, and 9 out of 103 (9%) respondents using unspecified persons for the task [25]. Thus, there is potential in technology such as digital images [26] to increase accuracy and objectivity in BCS compared to the traditional methodology of time consuming and subjective observer dependent manual scoring [27]. Recently, a variety of solutions for improved body condition scoring have been developed and tested, such as thermal imaging [28], the utilization of Fourier descriptors using still photos [29], machine learning [30], and 3D technology [31,32]. However, the above-mentioned systems are yet to be fully automated. Regardless of the technology used, a high degree of user-friendliness and easy implementation is paramount to attract mainstream cattle producers wishing for increased standardization and objectivity in their herd BCS assessments without the limitation of technical expertise. Finally, it is of key importance that commercially available BCS systems are field-tested properly to ensure that producers are provided with accurate data for their decision-making. Thus, the objective of this study was to validate a fully automated BCS camera system in comparison to manual body condition scoring in a commercial production setting. 

## 2. Materials and Methods 

The data collection took place at a commercial dairy farm in Greensburg, Indiana, on 9 April 2017. The farm housed approximately 3200 lactating and dry Holstein-Friesian cattle. All cattle were housed in groups based on the stage of lactation and milk production. Out of the farm population, 521 lactating cattle were scored once within each lactation group and the amount of cows scored per lactation group was determined from stratifying the proportion of the group in relation to the entire farm. BCS cameras (DeLaval body condition scoring BCS, DeLaval International AB, Tumba, Sweden) were mounted on the sort-gate at each exit (n = 2), where cows passed through daily post-milking. As the cow passed under the mounted camera a continuous video (30 FPS, 32,000 captured reference points) was taken and a 3D image from the video was automatically created and saved by the BCS camera software. In a secondary step, the saved 3D images were processed through an algorithm and analyzed to locate the key physical characteristics (pins, tail head ligaments, thurl, sacral ligaments, short ribs, and hooks) of the cow to calculate the automated score, viewable in DelPro Farm Manager (DeLaval International AB, Tumba, Sweden). The algorithm is based on the BCS scoring proposed by earlier studies but was reported in 0.1 increments in this study [1,33]. Details regarding the algorithm are considered a trade secret and not available for publication at this point. All automated BCS data were recorded in and downloaded from DelPro Farm Manager. To obtain manual body condition scores, three experienced staff members scored 343 cows, without communicating with each other, on the same day as when the automated scores were obtained, using a 1 to 5 BCS scale with 0.25 increments, as described in earlier studies [1,33].

### Statistical Analysis 

All statistical analyses were performed using SAS 9.3 (SAS Institute Inc., Cary, NC, USA). The manually scored BCS data set (MAN) consisted of cows where ≥2 out of 3 manual scores were within 0.50 of each other and averaged for a final averaged BCS per cow (n = 343). If one scorer was more than 0.50 off the other two scorers’ value, the average of the two remaining scorers would be assigned to the cow. The PROC FREQ function was used to assess frequency of agreement between scorers. Cows were removed from the study if they had only one manual score or no automated score. Interobserver reliability was determined using a Pearson correlation and Cohen’s Kappa coefficients (κ), using PROC CORR and PROC FREQ, respectively. 

Differences between the automatic and manual BCS were determined using a Pearson correlation. Cohen’s kappa coefficients thresholds were considered as follows: none, 0.00 to 0.20; minimal, 0.21 to 0.39; weak, 0.40 to 0.59; moderate, 0.60 to 0.79; strong, 0.80 to 0.90; and almost perfect, 0.90 to 1.00. Pearson correlation thresholds were considered as follows: negligible, 0.00 to 0.29, low, 0.30 to 0.49; moderate, 0.50 to 0.69; high, 0.70 to 0.89; and very high, 0.90 to 1.00. Descriptive statistics for all manual and automated scores was examined using PROC UNIVARIATE. Additionally, the automatically obtained BCS scores were structured into a continuous (0.10 increments) and a categorical data set with 3 body condition levels (low = BCS < 3.0, normal = BCS 3.0 to 3.75, and high = BCS > 3.75) to compare with the manual scores using 0.25 increments (manual continuous scores) or manual categorical scores (low = BCS < 3.0, normal = BCS 3.0 to 3.75, and high = BCS > 3.75). The rationale behind the categorical score thresholds was based around the observed means of the cows at the time of scoring (3.39 ± 0.32 and 3.27 ± 0.27 (mean ± SD) for manual and automatic scores, respectively). A t-test (PROC TTEST) was used to distinguish differences between the automated categorical scores and the manual BCS. To compare the manual and automated scores, plots of the differences between the automated and manual measurements were constructed against the calculated means of the manual and automated scores based on the methodology from earlier works [33,34].

## 3. Results

### 3.1. Interobserver Reliability

From the 343 scores utilized, at least two scorers scored within 0.25 increments of each other 69% of the time. The Pearson correlation coefficients between scorers had a strong, positive relationship. The correlations between the scorers were (r = 0.85) between scorers 1 and 2, (r = 0.87) between scorers 2 and 3, and (r = 0.86) for scorers 1 and 3. Weighted kappa coefficients between scorers were 0.62, 0.66, and 0.66; scorers 1 and 2, 2 and 3, and 1 and 3, respectively. 

### 3.2. Body Condition Scores

The mean (± SD) manual BCS score was 3.27 ± 0.48 (min = 2.25, max = 4.8) with a median (first quartile, third quartile) score of 3.3 (Q1 = 3.1, Q3 = 3.3). The mean automatic BCS scored by the camera was 3.38 ± 0.48 (min = 2.25, max = 4.8) with a median score of 3.4 (Q1 = 3.1, Q3 = 3.75). The Pearson correlation analysis showed that both the averaged continuous and categorical automated BCS scores were highly correlated with the manual scores, with a correlation of 0.78 and 0.76, respectively (Table 1). The continuous automatic BCS scores were equivalent to the continuous manual scores (Table 2). When looking at the categories of BCS, automatic categorical BCS scores were only equivalent to manual categorical scores in the BCS range of 3.00 to 3.75 (Table 2). The camera over-estimated the BCS from 44% of all cattle that were manually scored as lower than a BCS of 3.0 and under-estimated 92% of all cattle with a manual BCS of above 3.75 to be within the 3.0 to 3.75 range, while 5% of the cattle belonging 3.0 to 3.75 BCS range were incorrectly scored as under- or over-conditioned.

**Figure 1:**
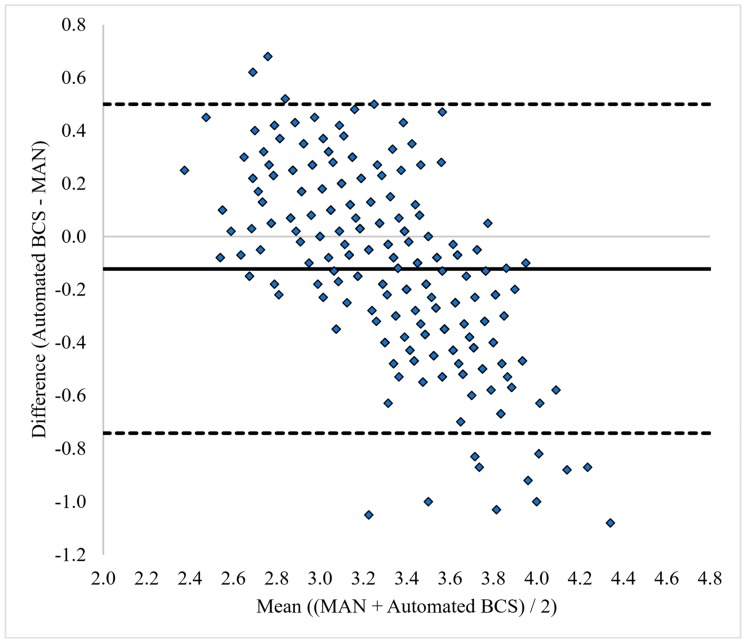
Bland–Altman plot of body condition scores (BCS) following [35] between manual average BCS scoring (MAN) and automated BCS scores. The solid line indicates the mean difference between the observation methodologies (Automated BCS – MAN) and the dotted lines represent 2 SD from the mean difference. The x-axis represents the range of the mean BCS for MAN and the automated BCS.

Bland–Altman plots of the agreement between manual scoring (MAN) and automated scoring are shown in Figure 1. The lowest mean difference (or highest agreement) between the manual scoring and the BCS camera was −0.12. The Bland–Altman plots demonstrated that the automated BCS camera tended to underscore cows as the BCS increased compared to either averaged manual scoring. 

## 4. Discussion 

The results from this study show that the implemented automated BCS camera system was able to identify the BCS of dairy cows equally accurate to manual scorers in scoring cattle in the categorical BCS range of 3.0 to 3.75, but it was inaccurate for cows with a BCS lower than 3.0 or above 3.75. In particular, the BCS of over-conditioned cattle was frequently placed into a lower BCS category by the BCS camera system. Similarly, under-conditioned cattle were placed into a higher BCS category by the BCS system compared to manual scorers. 

Overall, automated BCS and manual scores were correlated, but when broken down into BCS categories, strong correlations were only found between observations for the BCS range of 3.0 to 3.75. It is also possible that the key focal points of the cows’ body used by the system algorithm for scoring are either more or less distinguished in under- and over conditioned animals, respectively, resulting in small miscalculations of the BCS of individual animals outside of the more common BCS range of 3.0 to 3.75. This hypothesis is to some degree supported by the offset observed in the Bland–Altman plot (Figure 1) which demonstrates a higher degree of scoring difference compared to manual scorers with lower or higher BCS. The results confirm the potential of automated BCS as shown more than 10 years ago, but we still have the difficulty of accurately scoring body condition in dairy cattle using automation.

In our study, the correlation between the average continuous automatic and manual BCS was 0.78 while the correlation between the average categorical automatic and manual BCS was 0.76. These correlation coefficients are similar to recent study findings using a 3D Kinect camera (0.76) [32], but they are higher compared to a study comparing BCS derived from estimations of cattle body contours of thermal imaging (0.31) [36] and lower compared to later work optimizing the technique of thermal imaging (0.94) [28]. Thus, regardless of technology, the evolution of the automation process includes optimization, algorithm development, and software upgrades based on data derived from the field.

Our results show that the automated BCS is a reliable measure for on-farm management decisions. The benefits of readily available body condition scores cannot be overstated for producers when making management decisions. Producers can incorporate BCS into their management practices to provide useful information about nutrition status for individual cattle at different points in lactation. The implementation of a commercially available BCS camera system will initially be an expense for the producer but may have other positive economic implications on top of cutting cost on training and labor spent collecting BCS herd data. For instance, a stochastic simulation showed that specific herds could benefit from an automated system, such as a herd looking to improve reproduction efficiency [23]. The potential benefits varied based on overall herd BCS, but an automated BCS system may have additional positive economic impact regarding culling. The positive economic component is important as producers are more likely to adopt new technology if there is a high benefit to cost ratio [24]. 

An automated BCS camera with good accuracy and precision would allow for daily, consistently reliable information with no need for manual training or significant time commitment. We found that the automated BCS was consistent in scoring cattle from low to high BCS. Producers can analyze information provided by the technology such as changes in condition over time. Additionally, technology reduces human faults that may occur, such as incorrect scoring, incorrect data input, bias, and repeatability error.

The importance of a well-functioning BCS system is of even higher importance to producers with large herds that may only be able to score portions of cows each session due to time and labor constraints, while an automated system would have the potential to score the entire herd on a daily basis, leaving additional valuable time for other important tasks. Since small herds are subject to similar BCS errors as large herds, an automated BCS scoring system using cameras would be flexible enough to scale up to accommodate producers of variable farm size. Cattle that experience a low BCS between days 40 and 60 days in milk (DIM) correlate with lower reproductive success [14]. By monitoring BCS, producers will be able to make sound decisions regarding their reproduction programs. For instance, underweight and overweight cattle are more likely to experience dystocia, a condition that may be reduced with proper BCS management [37]. Additionally, high producing cows have traditionally been thinner and produce more milk directly from feed rather than fat reserves, making them more efficient, and making it important to maintain high producing cows at a healthy BCS [38]. By monitoring changes in BCS throughout lactation, producers can make important decisions for individual cow management as well as make assumptions on milk yields in the next possible lactation and schedule culling for low producing animals [39].

Cattle that show consistency in BCS change over multiple parities and, paired with milk yield records, can be selected for replacement. By creating a more accessible way of monitoring BCS, producers can utilize this tool to make genetic decisions within their herd through the selection of replacement heifers. Additionally, by identifying and recording patterns in BCS, we can select for patterns that are more desirable in the future, as a thorough analysis of BCS patterns may provide a new way to select for milk fat and protein [40,41].

## 5. Conclusions

To our knowledge, this study was the first evaluation of an implemented commercialized BCS camera system using 3D technology in field conditions. The BCS camera system was reliable for cattle that scored within the range of 3.00 to 3.75, where most cattle on the tested farm belonged, but did not score accurately within the other two BCS ranges of less than 3.00 and above 3.75. Cattle most often fall within the middle range, yet there is a need for adjustments to make the technology suitable for all body types. The advantage of using a BCS camera system with 3D technology is the opportunity to automatically select an optimal scoring angle from a continuous video sequence using a predetermined algorithm incorporated in the systems software. In addition, a BCS camera system using 3D imaging operates using a single camera compared to 2D still imaging cameras that use two cameras that need to work in synchrony, which provides one static view dependent on the cow body location, distance to cow, and body size of the cow in the taken image. The 3D technology also ensures that proper BCS scoring can be made regardless of the cow standing still, walking, or running past the camera. An automated BCS system may assist producers by ensuring less chance of human error, minimal labor, and provide readily available data for herd management decisions. This technology has the potential to serve as a reliable source of BCS scores, which can be incorporated into management practices. Automated BCS technology will allow producers to get more accurate and efficient BCS evaluations on a herd level compared to the more subjective, labor intensive, and error prone manual scoring.

## Figures and Tables

**Table 1:** Pearson correlation for cattle body condition scores (scale 1 to 5, low to high, n = 343) for continuous and categorical automated camera scoring versus manual scoring averages (MAN).

Data Compared	*p*-Value	r	n
Continuous camera vs. MAN	<0.001	0.78	343
Categorical camera vs. MAN	<0.001	0.76	343

**Table 2:** Regression analysis of cattle body condition scores (scale 1 to 5, low to high, n = 343) for automated continuous and categorical (low = body condition scoring (BCS) < 3.0, normal = BCS 3.0 to 3.75, and high = BCS > 3.75) BCS versus manual average BCS (MAN).

Dataset	Assessment	n	Minimum Difference	Mean Difference	Maximum Difference	*p*-Value
Continuous vs. MAN	Equivalent	343	−1.05	−0.11	0.60	<0.0001
Continuous vs. MAN < 3.00	Not Equivalent	70	−0.22	0.21	0.68	0.056
Continuous vs. MAN 3.00 to 3.75	Equivalent	199	−1.05	−0.10	0.50	<0.001
Continuous vs. MAN > 3.75	Not Equivalent	74	−1.08	−0.54	0.10	1.000
